# Computing Molecular Devices in *L*.*major* through Transcriptome Analysis: Structured Simulation Approach

**DOI:** 10.1371/journal.pone.0148909

**Published:** 2016-02-22

**Authors:** Pruthvi Raj Bejugam, Shailza Singh

**Affiliations:** National Centre for Cell Science, Pune, India; Jamia Millia Islamia, INDIA

## Abstract

In the modern era of post genomics and transcriptomics, non-coding RNAs and non-coding regions of many RNAs are a big puzzle when we try deciphering their role in specific gene function. Gene function assessment is a main task wherein high throughput technologies provide an impressive body of data that enables the design of hypotheses linking genes to phenotypes. Gene knockdown technologies and RNA-dependent gene silencing are the most frequent approaches to assess the role of key effectors in a particular scenario. Ribozymes are effective modulators of gene expression because of their simple structure, site-specific cleavage activity, and catalytic potential. In our study, after an extensive transcriptomic search of *Leishmania major* transcriptome we found a Putative ATP dependent DNA helicase (Lmjf_09_0590) 3’ UTR which has a structural signature similar to well-known HDV hammerhead ribozyme, even though they have variable sequence motifs. Henceforth, to determine their structural stability and sustainability we analyzed our predicted structural model of this 3’UTR with a 30ns MD simulation, further confirmed with 100ns MD simulation in presence of 5mM MgCl_2_ ionic environment. In this environment, structural stability was significantly improved by bonded interactions between a RNA backbone and Mg^2+^ ions. These predictions were further validated *in silico* using RNA normal mode analysis and anisotropic network modelling (ANM) studies. The study may be significantly imparted to know the functional importance of many such 3’UTRs to predict their role in a mechanistic manner.

## Introduction

Regulatory non-Coding RNAs have gained a lot more attention in the field of modern molecular biology in general and gene regulation either at pre transcriptional or post translational level in particular. As most of the well-studied organisms such as model organisms and infectious pathogens, genome wide analysis studies are increasing at fast pace with the advent of modern genomics and genome sequencing studies. These studies helps to unravel the underlying mechanisms of various biological processes such as the unknown mysteries behind certain pathogens pathogenicity, their survival even after the availability of the advanced chemotherapy and may help us to design effective vaccination.

One such kind of protozoan parasite which is highly prevalent in case of sub-tropical regions is *Leishmania*. Various species of this parasite causes various forms of leishmaniasis, which is the third most prevalent infectious disease in the world. As per 2010 statistics of World Health Organization (WHO) almost 2 million people suffer from various forms of this disease. The typical lifecycle of this parasite contains two hosts one is a vertebrate mostly human and other is an invertebrate host mostly a sand fly known as *Phlebotomus*. The major incident of leishmaniasis caused by *Leishmania major* is its cutaneous form. As these parasites are eukaryotes, they have a complex genomic structure with 36 chromosomes and most of these genes are arranged in head to head and tail to tail manner. Even though the genome of *L*.*major* was sequenced back in 2005[1] still a large chunk of information is missing from the regulation of many genes at key phases of its life cycle due to the lack of much needed transcriptome and proteomic data of various Leishmania sps.

Gene regulation at either the post transcriptional level or pre transcriptional level is a vital biological process. Controlling specific gene expression at various stages of the pathogen life cycle ultimately decides the fate of the pathogen. Biochemical and metabolomics [2] studies show many targets which can be aimed to control the pathogenesis at various stages.

*Leishmania* parasite regulates its gene expression in response to changes in their environment. The size of *L*. *major* haploid genome[1] is 32.8 Mbp which comprises of nearly 8,370 protein coding genes, 39 pseudo genes and 911 RNA genes approximately. In 8,370 genes only 36% have a putative function. The *L*. *major* genome predicts that protein coding genes are organized as large polycistronic units in a head to head or tail to tail manner. We all know that RNA polymerase II transcribes long polycistronic messages in absence of defined RNA polymerase II promoters, as a result of polycistronic transcriptions. mRNA synthesis requires host transcriptional control which involves 5’trans-splicing of a 39 nucleotide capped leader RNA and 3’cleavage of poly adenylation tail. Several examples of *Leishmania* support the notion that the developmental regulation of mRNA level is determined post transcriptionally by the sequence located in 3’UTR which usually controls mRNA stability and translation.

The post translational regulation is generally associated with stage specific gene association. Lack of promoter elements for RNA Pol II and unusually long 3’ UTR sequence provides the molecular basis of this type of control. Stage specific post transcriptional regulation is complex and involves the coordination of different mechanisms that can be independently triggered by environmental signals including the differentiation of promastigotes to amastigotes within mammalian macrophages.

Protein coding regions in *Leishmania* are not encoded by any intron with a single exception of gene encoding poly A polymerase which has reported the grouping of genes together into long strand specific poly cistronic clusters that are co- transcribed to yield poly cistronic mRNA. *Leishmania* seems to have lost the ability to regulate transcription initiation by RNA polymerase II and despite considerable efforts no canonical parameters for RNA pol II transcribed protein coding genes have been identified so far. Moreover the general transcriptional factors such as TATA box and TFIIB are highly divergent in Trypanosomatidae family. Recent reports suggests that *Leishmania* induces some non-coding RNAs which plays a vital role in its development and survival inside the host macrophage[3]. While in one report authors mentioned that *Leishmania* induced the repression of certain non-coding RNA elements which helped the host macrophages to polarize differently when compared with the normal ones[4], in another study novel class of regulatory non-coding RNAs were discovered regulating the development of this pathogen thus helping the parasite in its survival inside the host macrophage. [5]

The lack of control at level of transcription initiation might be explained by both the mode of poly cistronic transcription and the considerable need of these parasites during the complex life cycle, which may be more efficiently accomplished through downstream mechanism that involves RNA and protein regulatory processes. Numerous RNA binding proteins are encoded in the *Leishmania* genome, consistently with an active post transcriptional regulation of gene expression. The question is how the *Leishmania* RNA polymerase II transcribes genes in the absence of well-defined promoter elements[6]. It might be attributed to the organization of protein coding genes as a part of poly cistronic transcriptional initiation which is not a significant rate limiting factor in mRNA production. In the post genomic era scientists identify potential drug targets using bioinformatics approaches along with expression profiling and other tools to correlate gene expression with disease phenotype[7]. But the validation of these potential targets requires rapid techniques which are applicable to most genes and most cell types. Knockout mice play a vital role in validation process, however their production is expensive and time consuming. In contrast, gene specific silencing at mRNA level in cultured cells is an inexpensive rapid technique that can be adapted to high throughput strategies, wherein ribozyme technology [8,9] plays a remarkable role. Taking all these things into consideration we have adapted the approach laid down in this paper to know more about the non-coding segments regulatory effects in leishmanial transcriptome.

In our study we found a 3’ untranslated regions (UTR) present in the putative ATP dependent DNA Helicase (Lmjf_09_0590). This UTR is classified as Musashi Binding Element (MBE). It has specific RNA motif (ACCUUCUG) and helps in the formation of stem loop like secondary structure. This element is typically found in 3’ regions of the mature transcripts. It was initially discovered in *Xenopus* embryos and they help in the maturation [10] of those embryos. They specifically binds to Musashi protein in oocytes of *Xenopus* and helps in mRNA maturation [11]. In pursuit of this ultimate aim it can be said that this element may have a possible role in gene regulation at post transcriptional level.

## Materials and Methods

### Transcriptome Analysis of *Leishmania major*

Entire *Leishmania major* genome[1] was obtained from NCBI genome comprising of 36 chromosomes of *L*. *major* and were searched for total RNA sequences using BLASTN (NCBI) tool. RNA sequences of predicted and hypothetical protein genes were removed. Later, all these RNA sequences retrieved were checked for the regulatory elements like Riboswitches and 3’ and 5’ UTR sequences using RegRNA 2.0.[12]

### Structural predictions of the putative regulatory elements

Among the detected 3’UTR sequences, ATP dependent DNA helicase (Lmjf_09_0590) was predicted for secondary structures using Mfold[13] and RNAfold [14,15] programs. Mfold and RNAfold programs were automated through a set of python scripts (scripts provided as separate file). For Vienna format generation of secondary structure information of 3’ UTR sequences MC Cons [16] program was used. For tertiary structure predictions MC-Fold and MC-Sym[16] algorithms were used. To automate the process we have developed in house Ruby Scripts (developed and run on Ruby on Rails platform) which helps to generate the 3D structure models automatically. The generated structural models were statistically validated using Interactive Network Fidelity (INF)[17] value calculations, also called as the Matthew’s Correlation Co-efficient (MCC). INF can be calculated from the Positive predicted value (PPV) and Sensitivity (STY). PPV and STY are the relative terms determined by relative positive and negative predictions (i.e. True and False positives as well as negative values) as mentioned in Eqs (1) and (2)

$$PPV=\frac{TP}{TP+FP}$$ (1)

$$STY=\frac{TP}{TP+FN}$$ (2)

From Eqs (1) and (2)

$$INF=\sqrt{PPV\times STY}$$ (3)

### RNA Deformation Profile (DP) Analysis

Predicted putative 3’ UTR structures were subjected to Deformation Profile (DP)[17] analysis using DP matrix generation scripts generously provided by Prof. Eric Westhof (CNRS, France). These scripts use RNA 3d structures as their input and generate the matrices which when plotted as heat map, shows the deformation index of specific bases present in the given structure. Deformation index is a measure of total degree of discrepancies and the total amount of correctness in the predicted RNA structures. Deformation index can be calculated using RMSD and RNA Interactive Network Fidelity (INF), derived as discussed below:

If we compare between 2 given RNA structures, in which one is known and the other is a predicted one then RMSD and INF of both can be calculated using the above Eqs (1) and (2). From them deformation index can be calculated as

DI(A,B)=RMSD(A,B)/INF(A,B) (4)

### Molecular Dynamic Simulations (MDS) of the predicted RNA regulatory elements

After structure prediction and statistical validation of ATP dependent DNA helicase, the most favorable 3D structure model was of this sequence which was further checked for their stability by performing long range MD simulations. MD simulations were performed using Desmond 3.2 (D.E. Shaw Research). Updated AMBERf99 force field [18,19] (which was later reparametrized with α/γ [20] and χ_OL3_ [21]) was incorporated into Desmond using Viparr.py script. TIP3P water model was used in the orthorhombic box with a default 10nm cutoff PBC. Default long range and short range parameters were used along with a 2000 round of steepest descent minimization and a proper NPT and NVT equilibration were also performed. After equilibration a production MD run was performed with a time step of 2fs and a time scale of 30ns. All essential parameters such as potential energy, pressure, temperature were checked throughout the length of the MD simulation. After MD simulation RMSD, RMSF and other parameters of the trajectories were also calculated using default Desmond event analysis protocols and minimum energy structures were exported from the trajectories for further analysis.

### RNA Normal Mode Analysis

Normal mode is a pattern of motion in which all parts of the system move sinusoidally with the same frequency and with a fixed phase relation. In biological macromolecules such as proteins and RNA this can be calculated using the phase planes of the given system[22,23]. In our study normal mode analysis was performed using the ‘Phaser’[24] Module of PHENIX[25] software suite.

### Molecular Dynamic Simulations (MDS) of Putative Ribozyme in presence of MgCl_2_

It is a known phenomenon from earlier reports [26,27] that ribozyme should have a Mg^+2^ ion in its active state in its active conformation. To check whether the predicted RNA regulatory element has similar property and shows an interaction with Mg^+2^ ion in its minimum energy confirmation and how this Mg^+2^ ion helps in its stabilizing the folding kinetics, we performed 100ns MD simulation in presence of 5mM, 10mM and 25mM MgCl_2_ concentrations using Desmond (D.E. Shaw Research) with same parameters mentioned earlier. All MD simulations were performed on HP ProLiant SL390s workstation equipped with 2 Intel Quad core processors and 1 Nvidia tesla M2090 GPUs with 4190 Mb memory. After successful completion of MD simulations all the essential quality parameters were analyzed and RMSD, RMSF were calculated along with the interactions of putative ribozyme and Mg^+2^ ions during the entire length of MD simulations. All the plots were generated using the Desmond 3.2 analysis tools. Structural analysis, images and movies were generated using Pymol (Delano Scientific).

## Results and Discussion

### Transcriptome Analysis and Regulatory Element Prediction

Entire Genome of *L*.*major* (36 Chromosomes with 8272 protein coding genes, 911 RNA genes & 39 pseudogenes) were used to extract the transcriptome using NCBI BLASTN tool. Resulting transcriptome was searched for regulatory elements such as Riboswitches and UTR sequences using RegRNA 2.0. 340 regulatory elements were extracted from 3’UTR regions of many RNAs.

### Secondary and Tertiary Structure Prediction of Regulatory elements

**Fig 1:**
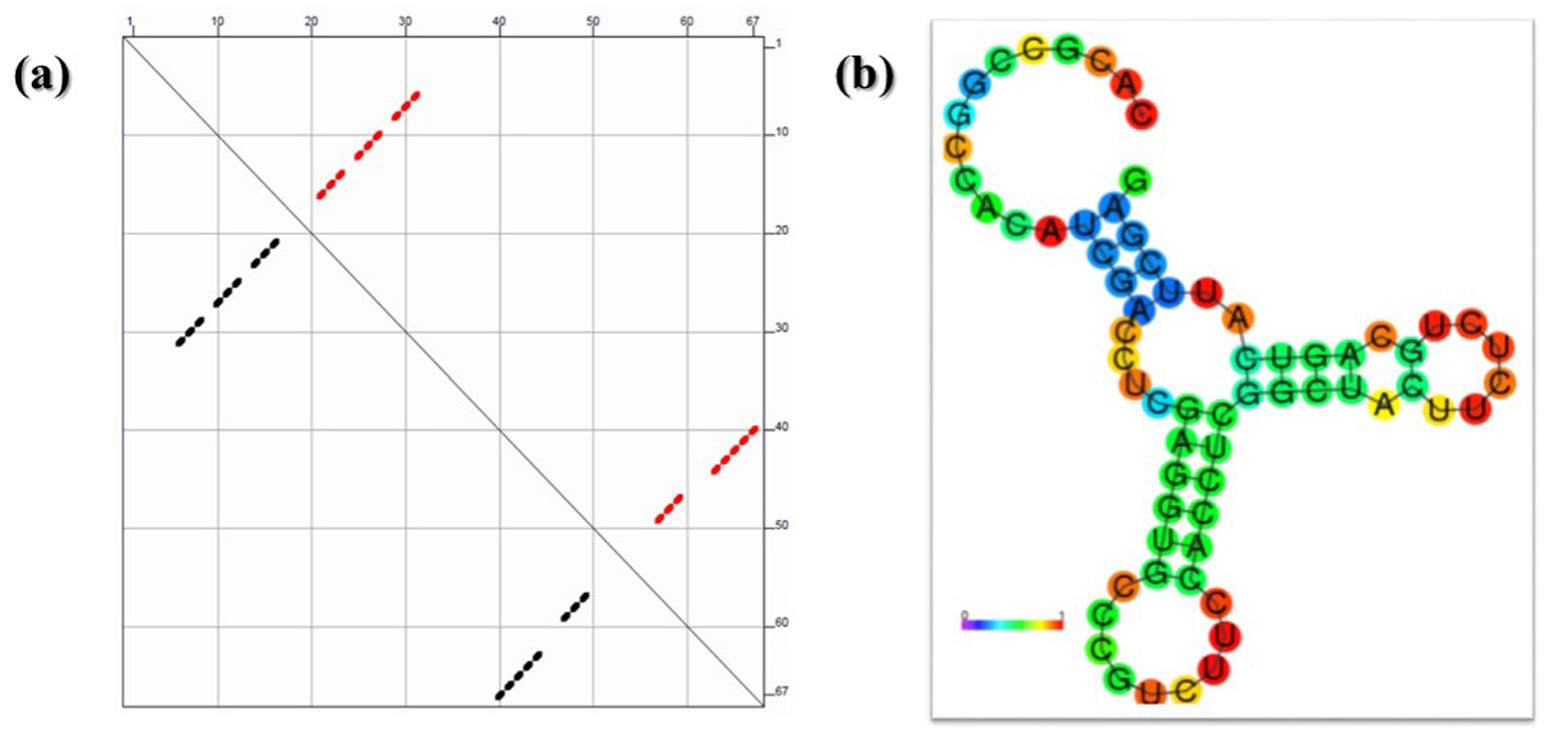
Secondary structure prediction of ATP dependent DNA helicase 3’ UTR with calculated δG = -11.7Kcal.mol-1 in this (a) is a graphical representation of each base pairs minimum free energy (MFE) and (b) is secondary structure of the predicted 3’UTR showing the proper formation of stem loop like structures with a typical hammer head like structure.

**Fig 2:**
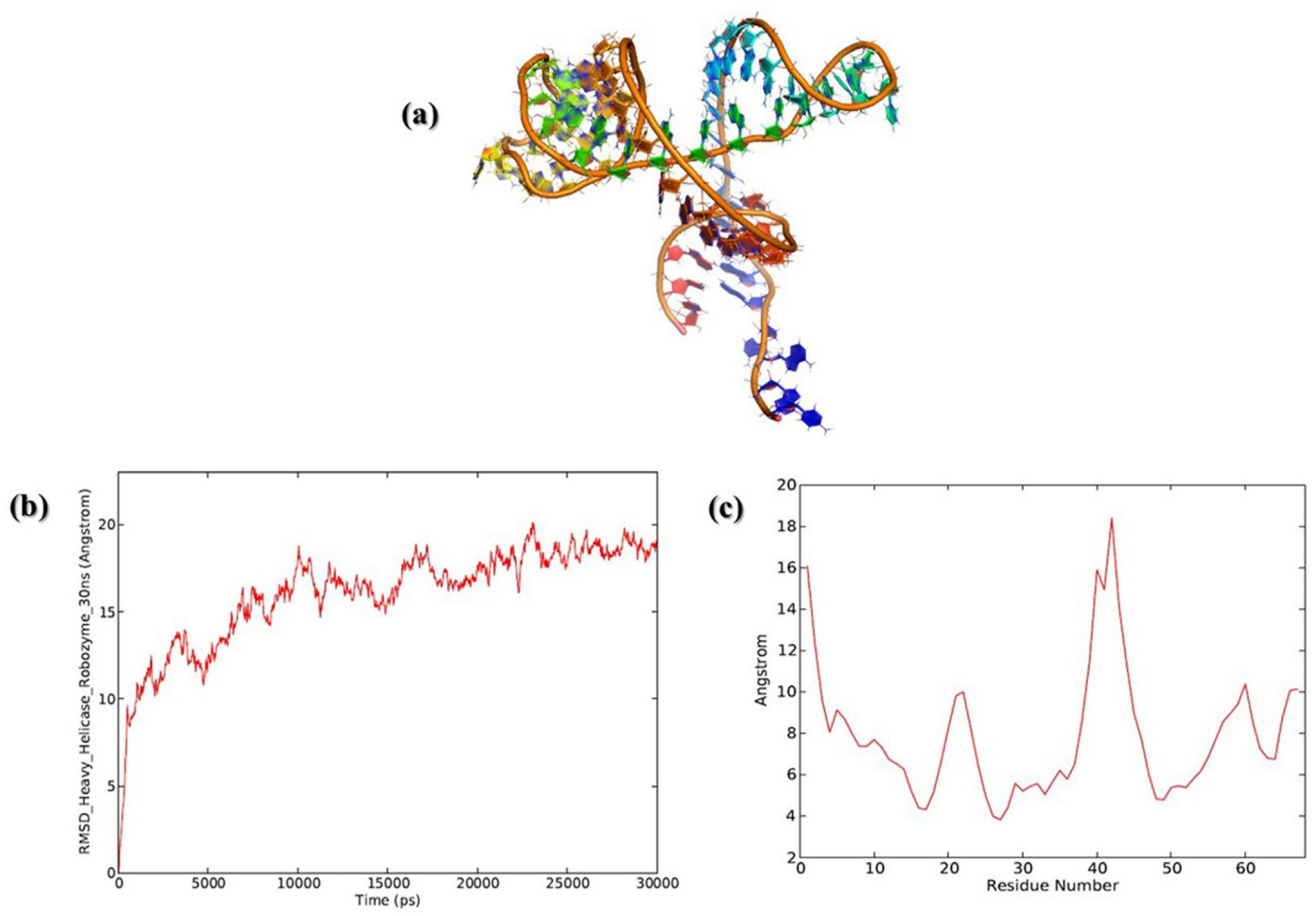
Tertiary structure predictions of ATP dependent DNA helicase putative ribozyme with calculated δG = -14.2 Kcal.mol^-1^ (b) RMSD and RMSF plots of 30ns MD simulation ATP dependent DNA Helicase putative Ribozyme trajectory. Left panel shows the RMSD and after 6 ns MD simulation the trajectory seems to be stabilized (c) Right panel shows the RMSF indicating that bases 19–22 and 37–42 are highly flexible regions of putative ribozyme.

340 regulatory sequences were used for 2D structure predictions using M-fold and RNAfold algorithms. After secondary and tertiary structure predictions, 3’ UTR ATP dependent DNA Helicase (Lmjf_09_0590) has a similar structural signature of a typical Hammerhead Ribozyme and Hairpin loop Ribozymes. The 3’ UTR has nearly 60–70 bases with a signature of Musashi Binding element (MBE). The structural predictions were statistically validated using Interactive Network Fidelity (INF). All structural models of 3’UTR elements have more than 90% of INF which is considered desirable for the accurate RNA structural predictions. Values are tabulated and provided in S1 Spreadsheet. Along with the other statistics such as Deformation indices (DI), Fig 1 shows secondary structure predictions using Vienna RNAfold algorithms and the MFE diagram indicates most of the secondary structure has calculated δG = -11.7 Kcal.mol^-1^. After tertiary structure prediction δG was further reduced to -14.2 Kcal.mol^-1^ and the best predicted 3d structure has mean RMSD of 1.28 Å with the other set of predicted models. This model has an overall INF of 95.1% suggesting the model to have the highest accuracy. ATP dependent DNA helicase putative ribozyme 3’ UTR was similar to the Hammerhead Ribozyme structure of Hepatitis Delta Virus (HDV) trans acting ribozyme[26,27]. The predicted 3d models have the canonical, non- canonical base pairing essential for RNA structural stability and integrity. The predicted and energy minimized tertiary structure of this 3’ UTR was shown in Fig 2a.

### Molecular Dynamics (MD) Simulations

**Fig 3:**
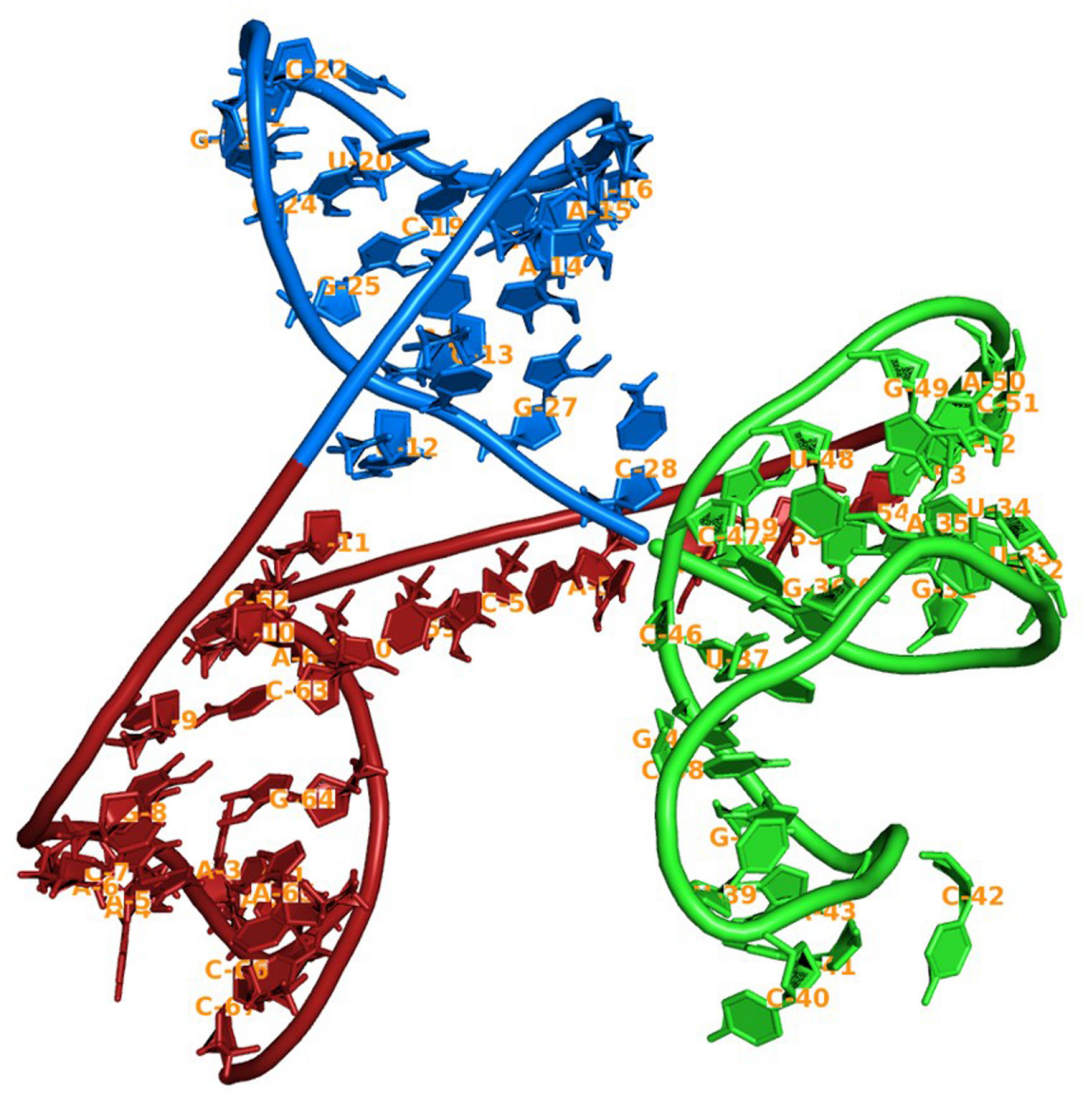
Rigid domains of Putative Ribozyme identified using RNA Normal Mode analysis indicated with red and blue colors, flexible domains are denoted in green color.

**Fig 4:**
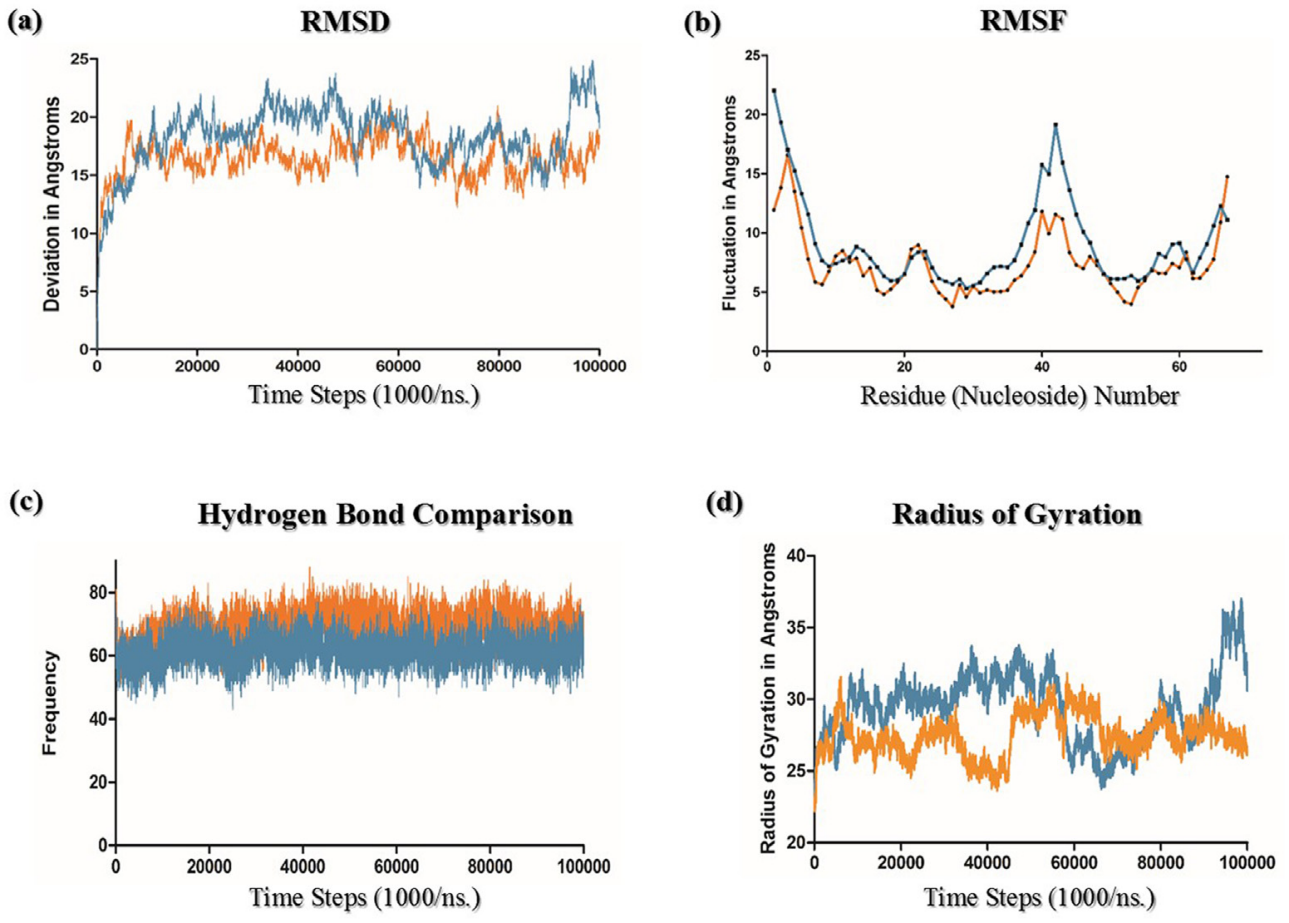
MD simulation event analysis comparison of Helicase 3’ UTR 100ns MD simulations in presence and in absence of MgCl_2_. In all the images events of Helicase 3’ UTR is represented in light blue color and the same events in presence of MgCl_2_ is represented in orange colour (a) RMS deviation is more stabilized in presence of MgCl_2_ than in the absence of salt. (b) RMS fluctuations are recorded similarly when compared with the presence of salt showing the lesser fluctuations than in its absence. (c) No. of. Intra molecular Hydrogen bond analysis suggests that in presence of salt RNA is more stable than in the absence of it. (d) Radius of gyration analysis also shows more stable structural movements in presence of salt rather than in its absence. Thus our simulations in presence of MgCl_2_ shows more favorable environment rather than in the absence of it.

MD simulation for ATP dependent DNA helicase was performed for 100ns and the trajectory was stabilized after 6ns of MD production run. Simulation event analysis indicated that all the parameters are maintained constantly throughout the simulation time length visualized in Fig 2. RMSD and RMSF are calculated and they show a stable trajectory. Stable trajectory minimum energy conformation of ATP dependent DNA helicase putative ribozyme was exported and used for further analysis. RMSD and RMSF plots are described in Fig 2. RMSF analysis suggested that regions 19–22 and 37–42 are showing the highest fluctuations which are indicative of the fact that they might be the critical regions of this putative ribozyme. Fig 3 depicts rigid domains of putative ribozyme predicted through Normal Mode Analysis. During trajectory analysis of MD simulation proper folded and unfolded states were observed. In MBE element structural motif showed the least dynamics and their structural signatures are maintained during the entire course of MD simulation. Entire trajectory is graphically represented in S1 Movie. The hydrogen bonds between both canonical Watson—crick base pairing as well as Hoogsteen base pairing can be visualized in the movie. Hydrogen bonds are shown as red dashes in the S2 Movie. Further this MD simulation is extended to 100ns time scale and the events are analyzed and plotted in Fig 4. Major events such as RMSD and RMSF are shown Fig 4a and 4b. Intra molecular hydrogen bond analysis indicates that throughout the length of MD simulations structural folds are maintained constantly represented in Fig 4c. Radius of gyration is shown in Fig 4d, indicates that throughout the total time scale of MD simulation structural fluctuations allowed Helicase 3’ UTR to attain a more stable conformation.

### RNA Deformation Profile Analysis

While performing the deformation profile analysis a standard hepatitis delta virus (HDV) ribozyme (PDB Id: 4PR6) is used as a reference structure and after obtaining their deformation indices (DI), their Di’s were plotted against each other. We found most of the nucleosides are with proper Di’s with lower or optimized energies. Some of the terminal bases have the higher DI with higher energy. Detailed DI values of the top 30, 3d structural models are presented in the S1 Spreadsheet.

### RNA Normal Mode analysis

After successful NMA, two rigid normal mode domains are identified from the putative ribozyme shown in Fig 3. The first rigid domain is observed from 12^th^ base to 28^th^ base and the second rigid domain is observed from 29^th^ to 58^th^ bases. Rest of the structure is shown as flexible. NMA indicates that rigid domains impart structural rigidity and firmness. Flexible regions may help in structural dynamics important for their activity. NMA is in corroboration with earlier MD simulation analysis performed on putative ribozyme, wherein most of the flexible regions show high degree of movements and rigid domains shows lesser fluctuations.

### MD simulation of the Putative Ribozyme in presence of MgCl_2_

In order to identify the conformational changes during the flexible state of the putative ribozyme, we performed MD simulations at various concentrations (5mM, 10mM and 25mM) of MgCl_2_. 10mM and 25mM MgCl_2_ system RMSD analysis shows an unstable RNA conformations, whereas 5mM MgCl_2_ salt concentration shows a stable RMSD, observed in S2 Fig, (RMSD of 5mM MgCl_2_ system is shown in S2A Fig, 10mM and 25 mM MgCl_2_ RMSD analysis is illustrated in same S2B and S2C Fig). 10mM MgCl_2_ MD simulation is further extended till 100ns time length. After successful completion of the simulation, trajectories are analyzed for their quality parameters such as pressure, temperature and volume apart from energy and potential energies of the system illustrated in S1 Fig. Quality analysis indicates that all the essential parameters are maintained constantly during the entire time scale and potential energy of the molecule indicates that it has been stabilized in presence of MgCl_2_.

RMSD and RMSF analysis shows that in presence of MgCl_2_, MgCl_2_ has stabilized the movements of the flexible domains identified from earlier RNA normal mode analysis and trajectory analysis. It also indicates that some of the Mg^+2^ ions interact with the active ‘Adenosine’ base which has stabilized the flexible domains as well as the less fluctuating site of MBE Box 37–42. RMSD and RMSF plots are shown in Fig 4a and 4b. Analysis of these plots suggests decrease in mean RMSD and RMSF value from the above normal 100ns MD simulation due to the presence of Mg^+2^ ions. Their interaction with the RNA backbone suggests that Mg^+2^ ions play a vital role in stabilization of catalytically active regulatory RNAs such as ribozymes. Trajectory analysis of 100ns MD simulation showed the improved stability and sustainability of the 3’ UTR during the entire MDS. Apart from the RMSD and RMSF analysis, we have also calculated the intra molecular hydrogen bonds which were formed during the entire time scale of MD simulation, represented in Fig 4c. These intra molecular hydrogen bonds indicate ATP dependent DNA Helicase 3’ UTRs tertiary structure has stabilized these stable hydrogen bonds. In presence of MgCl_2_ some of the Mg ^2+^ ions form hydrogen bonds with the backbone of certain of RNA bases (C 26) shown in S2 Fig. The entire trajectory is shown as an animation movie in S2 Movie. In this animation we observe the intramolecular hydrogen bonds as yellow dashes. They are the part of canonical and non-canonical Watson- Crick base paring as well as Hoogsteen base pairing. We can also observe some hydrogen bonds between Mg ^2+^ ions (Blue spheres in animation) and RNA backbone represented as yellow color dashes. Hydrogen bonds between Mg ^2+^ and RNA backbone may be the main cause of its improved stability and better dynamics suggesting Mg ^2+^ ions play a vital role in its catalytic activity. One of the hydrogen bond interaction between Mg^+2^ion and a nucleoside C26 of 3’ Helicase UTR was mapped using LigPlot+[28] program and shown in S3 Fig. Based on these predictions, statistical validation and molecular dynamics simulation analysis, we hypothesize that 3’ UTR (MBE) of ATP dependent DNA Helicase may have the catalytic activity and may act as hammer head ribozyme similar to HDV ribozymes [26,27].

**Fig 5:**
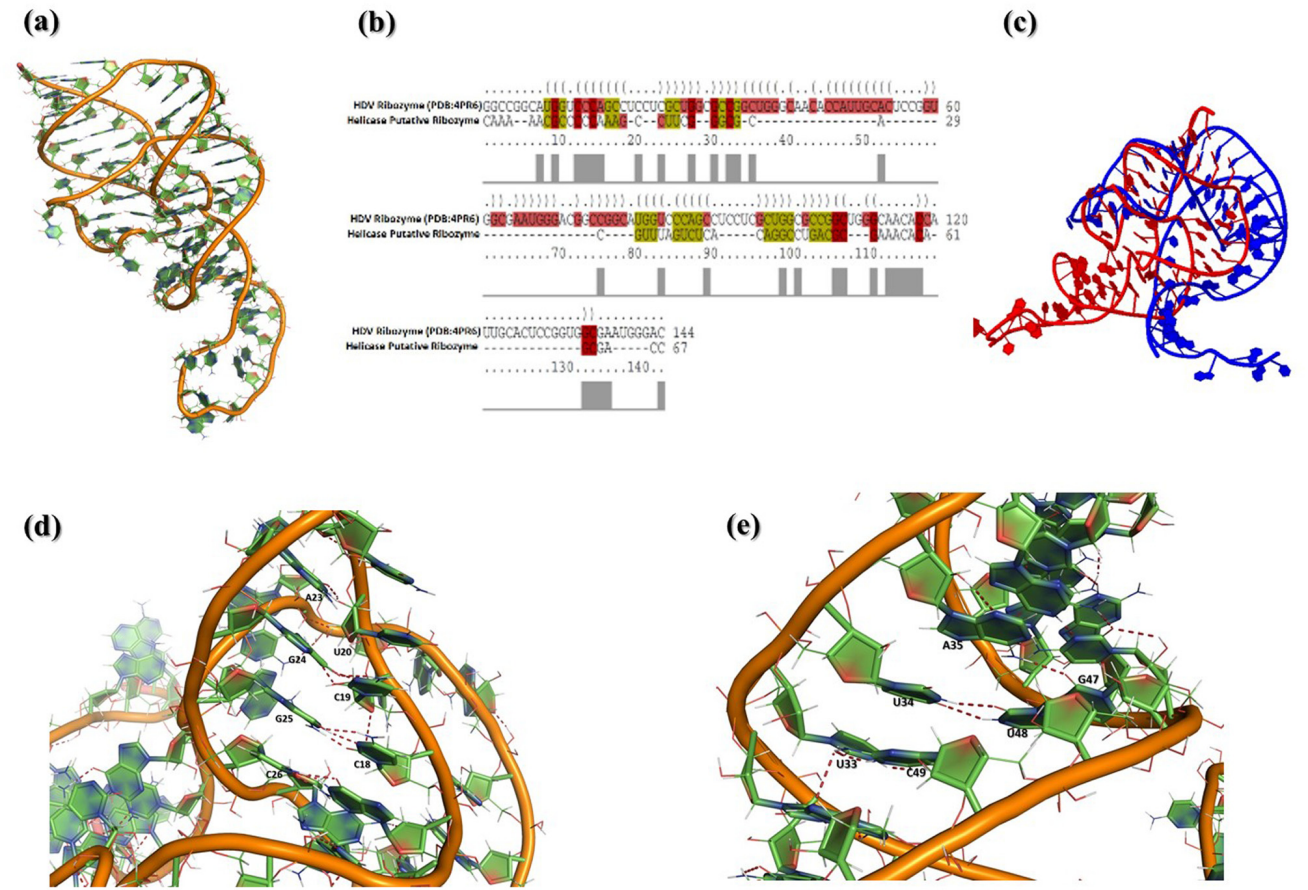
Comparative analysis of Helicase 3’ UTR with Hepatitis Delta Virus (HDV) ribozyme (PDB Id: 4PR6). (a) Tertiary structure of HDV Ribozyme (4PR6). (b) Sequence alignment of Helicase 3’ UTR and HDV ribozyme. (c) Structural superimposition of HDV ribozyme (4PR6) and Helicase ribozyme where HDV ribozyme is represented in red and Helicase 3’ UTR in blue color respectively. (d) Canonical base paring are observed during the MD simulation of Helicase 3’UTR. In this we have shown the base paring between G25-C18, A23-U20, which are regular Watson-crick base pairings. (e) Non-canonical base pairing which are normally seen in RNA structures are also observed during Helicase 3’ UTR MD simulations. In this we have shown base-pairings between A35-G47, U34-U48, and U33-C49. Apart from these Hoogsteen base parings are also seen.

Apart from MD simulation analysis, structural analysis of Helicase 3’ UTR minimum structure obtained from MD trajectory analysis depicts that it has many canonical and non-canonical interactions, such as base paring of G25-C18, A23-U20 are some of the canonical Watson-crick base paring and A35-G47, U34-U48, U33-C49 are non-canonical base parings represented in (Fig 5d and 5e). Moreover helicase 3’ UTR shows good structural similarity with HDV ribozyme (PDB Id: 4PR6) (Fig 5c) and thus it can be said that it has similar characteristics of a ribozyme. Alignment of putative ribozyme is shown with the HDV ribozyme (PDB: 4PR6) (Fig 5b).

### Conclusion

Gene specific regulation in parasites can be used extensively for the study of various metabolic processes under the user defined conditions which may enable the researchers to understand function of specific gene in presence or absence of another specific gene. This kind of scenario is very hard in case of the eukaryotic parasites such as Leishmania. We aim to create synthetic RNA mediated specific auto regulatory element which may be used for the above mentioned purpose. Artificial RNA regulatory elements were found during extensive analysis of entire leishmanial transcriptome. Further structural predictions and statistical validation suggested ATP dependent DNA Helicase 3’ UTR to be the putative ribozyme. Extensive structural analysis which includes MD simulations and MD simulations in presence of MgCl_2_ adds strengths to our initial predictions. These simulation studies help to understand the mechanistic behavior of these putative regulatory elements. Hammerhead like ribozyme structures suggested that these RNA regulatory elements might also act like autoregulatory ribozymes.

Although structural studies of regulatory RNAs are still in its infancy, one can foresee it to play a pivotal role in the regulation of gene expression involving long range structurally complex interactions. Elucidating the importance of structural states in RNA may help to discuss the mechanistic role of non-coding RNAs in disease, thus aiding in the creation of structural framework necessary for RNA-based therapeutics.

In future we would like to further validate the initial predictions using *in vitro* transcription assays, functional characterization using ribozyme cleavage assay and RNA EMSA. Further biophysical characterization would help us to create artificial gene specific autoregulatory elements and may even be used to deliver into *Leishmania* parasites using the liposomal formulations for therapeutic intervention. Development of ribozymes as innovative nucleic acid-based enzymes that have the capability of coupling a catalytic function to a molecular switch may certainly open the way to the development of artificial RNA—based networking systems which will be quite fascinating.

## Supporting Information

S1 Fig Quality analysis plot of 100ns MD simulation of ATP dependent DNA helicase putative ribozyme in presence of MgCl_2_. (a, b) Energy of the system and potential energy of the molecule indicate that during the simulation potential energy is decreased. (c-e) pressure, temperature and volume are constantly maintained in the entire 100ns MD simulation.
(TIF)

S2 Fig MD Simulationand RMSD analysis of 3’ Helicase UTR in presence of various MgCl_2_ concentrations. (a) RMSD analysis of 3’ Helicase UTR at 5mM MgCl_2_ concentrations. (b,c) RMSD analysis of the same at 10mM and 25mM salt concentrations.
(TIF)

S3 Fig Hydrogen bond interaction showed one of the nucleoside C26 of Helicase 3’ UTR and Mg^+2^ ion during the 100ns MD simulation. This hydrogen bond was observed from the one of the snapshots of MD trajectory and H-bond interaction was plotted using Ligplot+ program.
(TIF)

S1 Movie Movie shows the behavior of the putative Helicase 3’ UTR 30ns MD simulation. In this movie we can visualize the fluctuations observed at both 3’ and 5’ end, but the rigid domains identified from normal mode analysis shows constant 2D and 3D structures.
(MP4)

S2 Movie Movie shows the behavior of the putative Helicase 3’ UTR 100ns MD simulation in presence of MgCl_2_. During the MD run we have observed some of the Mg^+2^ ions are forming hydrogen bonds with some of the nitrogen bases. One such example is illustrated in Fig 5 of this manuscript. The stabilization of this RNA molecule may be attributed to the presence of Mg^+2^ ions and their interaction.
(MP4)

S1 Spreadsheet Supplementary spread sheet contains the statistics of top 30 models which includes 2D, 3D INF values and Deformation Index (DIs), RMSD etc. These values are calculated from the automated python scripts.
(XLSX)

## Abbreviations

MDS: Molecular Dynamics Simulation
INF: Interactive Network Fidelity
MCC: Mathews Correlation Co-efficient
PPV: Positive predicted value
STY: Sensitivity
DP: Deformation Profile
RMSD: Root Mean Square Deviation
RMSF: Root Mean Square Fluctuation
PBC: Periodic Boundary Condition
NMA: Normal Mode Analysis
HDV: Hepatitis Delta Virus
MBE: Musashi Binding Element
MFE: Minimum free Energy
