# Supplementary figures and images for: Computing Molecular Devices in L.major through Transcriptome Analysis: Structured Simulation Approach
Source: PLoS One. 2016 Feb 22;11(2):e0148909. doi: 10.1371/journal.pone.0148909 (PMC4768835; doi:10.1371/journal.pone.0148909)

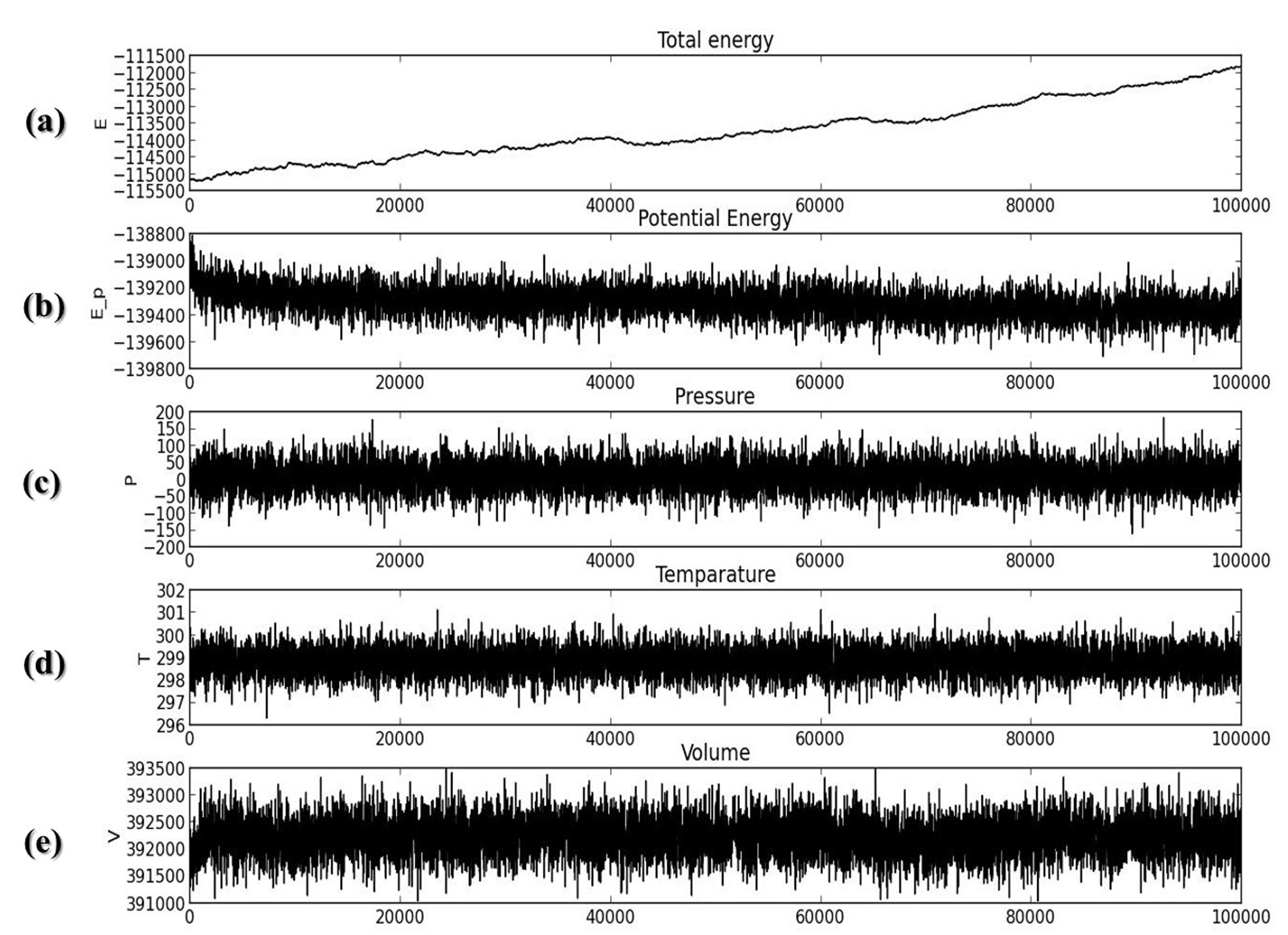

Supplement: S1 Fig — (a, b) Energy of the system and potential energy of the molecule indicate that during the simulation potential energy is decreased. (c-e) pressure, temperature and volume are constantly maintained in the entire 100ns MD simulation. (TIF) [file pone.0148909.s001.tif]

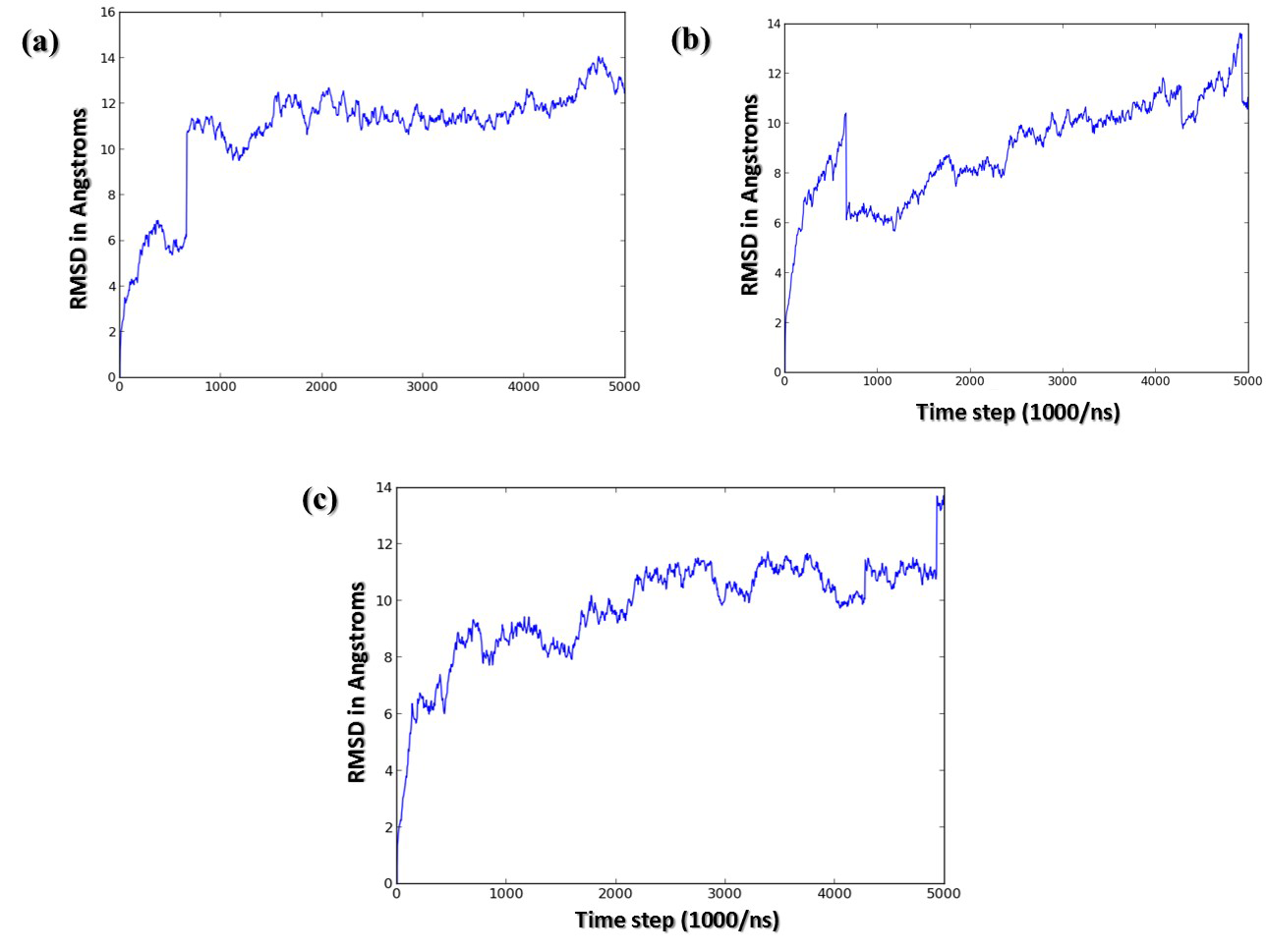

Supplement: S2 Fig — (a) RMSD analysis of 3’ Helicase UTR at 5mM MgCl2 concentrations. (b,c) RMSD analysis of the same at 10mM and 25mM salt concentrations. (TIF) [file pone.0148909.s002.tif]

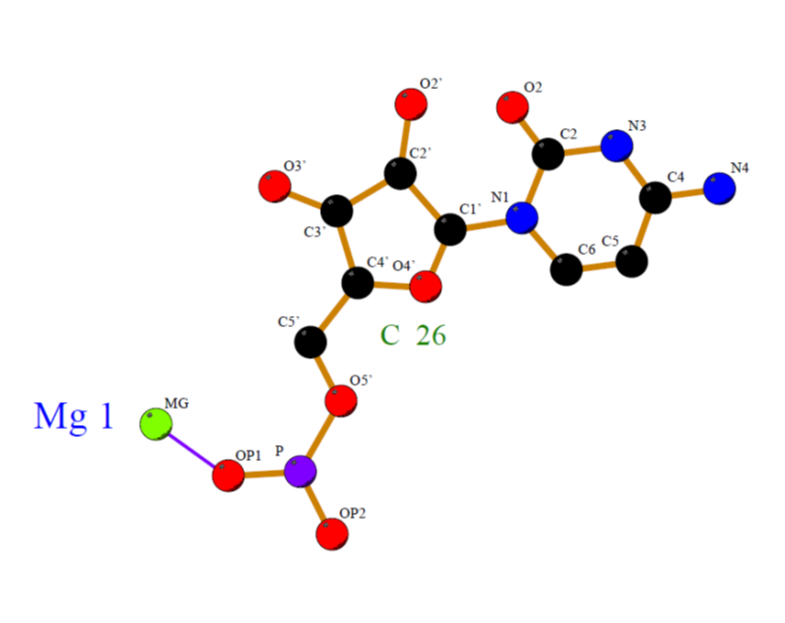

Supplement: S3 Fig — This hydrogen bond was observed from the one of the snapshots of MD trajectory and H-bond interaction was plotted using Ligplot+ program. (TIF) [file pone.0148909.s003.tif]
